# Asymmetrical Damage of the Wrist Joint Induces Lateralized Cortical Bone Loss in the Metacarpal Diaphysis in Patients with Rheumatoid Arthritis

**DOI:** 10.3390/jcm13247652

**Published:** 2024-12-16

**Authors:** Akikatsu Nakashima, Hiroshi Fujii, Masahiro Kuroda, Takeshi Zoshima, Ichiro Mizushima, Hideki Nomura, Mitsuhiro Kawano

**Affiliations:** 1 Department of Internal Medicine, Asanagi Hospital, 1-8 Gofuku-machi, Takaoka 933-0906, Japan; nakashima_skh@yahoo.co.jp (A.N.);; 2 Division of Nephrology and Rheumatology, Ishikawa Prefectural Central Hospital, 2-1 Kuratsukihigashi, Kanazawa 920-8530, Japan; 3 Department of Nephrology and Laboratory Medicine, Graduate School of Medical Science, Kanazawa University, 13-1 Takara-Machi, Kanazawa 920-8640, Japan; 4 Department of General Medicine, Kanazawa University Hospital, 13-1 Takara-Machi, Kanazawa 920-8640, Japan; 5 Department of Hematology and Immunology, Kanazawa Medical University, 1-1 Daigaku, Uchinada, Kahoku-gun 920-0293, Japan

**Keywords:** rheumatoid arthritis, metacarpal bone density laterality, asymmetric wrist joint damage

## Abstract

**Background/Objectives**: Osteoporosis is common in rheumatoid arthritis (RA), occurring either systemically or locally around inflamed joints. Decreased metacarpal bone density is a known marker of RA progression and hand function impairment. Although RA is generally characterized by symmetrical arthritis, some patients exhibit asymmetrical joint involvement. This study investigates the frequency of unilateral metacarpal bone density reduction in RA patients and aims to identify associated factors. **Methods**: This study included 143 RA patients (107 females, mean age 62.4 yrs., mean disease duration 11.1 yrs.). Bilateral hand X-rays were used to measure the cortical thickness rate (CTR) of the 2nd to 4th metacarpals. Unilateral bone density reduction was defined as a thin-to-thick-side CTR ratio (CTRR) < 0.8. Associations between CTR reduction and unilateral wrist joint damage (WJD) were analyzed. **Results**: Unilateral CTR reduction (CTRR < 0.8) was observed in 16.8% of patients, significantly associated with unilateral WJD. Among patients with unilateral WJD, 50.0% showed CTRR lateral (+) compared to 10.1% without unilateral WJD (*p* < 0.01). ANCOVA revealed significant effects of WJD laterality on CTRR, with an interaction effect showing greater CTRR laterality when thin-side WJD was present without thick-side WJD. Post-biologic treatment, CTR values decreased in both hands, indicating no improvement in bone density reduction. **Conclusions**: Approximately 17% of RA patients exhibited unilateral relative metacarpal bone density reduction, closely associated with unilateral WJD. This first detailed report on bone density laterality in RA underscores the need for early intervention and rehabilitation strategies in RA patients with hand involvement.

## 1. Introduction

Rheumatoid arthritis (RA) is a chronic inflammatory systemic autoimmune disease characterized by the inflammation of the synovial membrane. While the etiology of RA is unclear, environmental factors, genetic susceptibility, and interactions among multiple immune cells are assumed to be involved in RA pathogenesis [1].

In this era of biological agents, the prognosis of patients with RA has improved. However, the prevalence of osteoporosis in RA patients remains high. Osteoporosis in RA patients likely occurs through two mechanisms: generalized osteoporosis, observed as axial skeletal osteoporosis associated with factors such as immobility, glucocorticoid use, and other comorbidities [2,3]; and localized osteoporosis that develops around the joints due to inflammation and inflammatory mediators, which is observed as cortical bone loss in the diaphysis [4,5]. Research on osteoporosis in RA has mainly focused on generalized bone loss, with less attention to localized loss, seen as cortical reduction around the joints [6,7].

Periarticular osteoporosis, a form of localized osteoporosis, has been identified as one of the characteristics of RA [8,9]. Evidence suggests periarticular osteoporosis occurs early in the course of RA [10] and has been suggested to precede bone erosion [11]. Periarticular osteoporosis is thought to be directly influenced by inflammation, primarily driven by the local release of inflammatory cytokines [12]. Cross-sectional studies showed that bone loss in the diaphysis regions of hand cortical bones was associated with joint damage, suggesting a relationship between these processes. Therefore, periarticular osteoporosis has distinct risk factors compared to systemic osteoporosis, which is primarily induced by immobility and treatments such as glucocorticoids [13,14,15].

Regarding inflammatory bone loss in the hands, osteoporosis occurs not only in periarticular regions directly affected by arthritis but in the cortical bone of the metacarpal diaphysis. This suggests that inflammation-induced bone loss may extend to adjacent cortical bones even without direct synovial contact [16,17,18]. Furthermore, decreased bone density in the metacarpals has been reported as a surrogate marker for disease progression [19,20,21], morbidity [22,23], comorbidity [24,25], and impaired hand function [26], highlighting the importance of its assessment.

Symmetrical arthritis is characteristic of RA and was included in the 1987 Rheumatoid Arthritis Classification criteria [27]. It is defined as simultaneous involvement of the same joint areas on both sides of the body, where bilateral involvement of one or more proximal interphalangeal, metacarpophalangeal, and metatarsophalangeal joints is acknowledged without requiring absolute symmetry. However, studies show that some patients exhibit asymmetrical arthritis [28,29].

**Figure 1:**
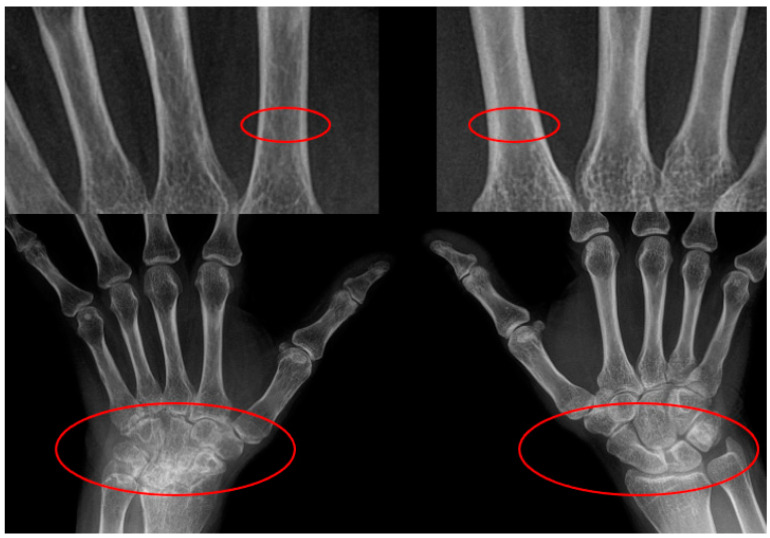
Rheumatoid arthritis patients typically exhibit symmetrical arthritis, though some are known to show asymmetric arthritis. We encountered a patient with asymmetrical osteoporosis of the metacarpal bones and asymmetrical carpal lesions, which inspired this study. Upper Red Circled Regions: These focus on the cortical bone of the metacarpal diaphysis, showing differences in cortical thickness. The thinning of the cortical bone in these areas is more pronounced on one side, suggesting localized bone loss, often associated with periarticular osteoporosis in rheumatoid arthritis. Lower Red Circled Regions: These highlight the wrist joint areas, illustrating bone erosion, joint space narrowing, or other joint damage. A clear asymmetry between the left and right wrist joints is observed, indicating more severe joint damage on one side, likely due to localized inflammation and progressive joint damage.

We encountered a case of asymmetrical osteoporosis in the metacarpals of a patient presenting with asymmetrical lesions in carpal bones (Figure 1). While decreased bone density in the metacarpal diaphysis is characteristic of RA-associated osteoporosis, whether or not asymmetrical hand arthritis leads to unilateral reduction in hand bone density remains unclear. Based on previously reported methods to evaluate the thickness of metacarpal cortical bone [25,30], we examined the frequency of unilateral cortical bone density reduction in the metacarpal diaphysis. We aimed to determine the frequency of unilateral bone density reduction in RA patients with asymmetrical hand arthritis and to identify contributing factors beyond joint damage that may underlie these differences.

## 2. Materials and Methods

### 2.1. Subjects

The study participants comprised 143 RA patients (107 females) diagnosed according to 1987 American College of Rheumatology classification criteria for RA [27] and/or 2010 ACR/EULAR classification criteria for RA [31] and treated at Ishikawa Prefectural Central Hospital or Kanazawa University Hospital. Although patients diagnosed before 2010 were initially classified using the 1987 criteria due to the unavailability of the 2010 criteria at that time, all patients met the 2010 ACR/EULAR classification criteria at the time of this investigation.

### 2.2. Methods

C-reactive protein (CRP), rheumatoid factor (RF), anti-cyclic citrullinated peptide antibodies (anti-CCP), and erythrocyte sedimentation rate were measured from patient serum samples. Swollen and tender joints, patient visual analogue score (VAS), and physician VAS were assessed during physical examinations. Based on these assessments, disease activity indices such as the Clinical Disease Activity Index, Simplified Disease Activity Index, Disease Activity Score in 28 joints with Erythrocyte Sedimentation Rate, and Disease Activity Score in 28 joints with CRP were calculated. In some patients, the Health Assessment Questionnaire was also evaluated. Smoking status, alcohol consumption, and comorbidities, including diabetes mellitus and chronic kidney disease (CKD), were assessed from medical records. Diabetes was defined as its diagnosis prior to or at baseline, or use of oral antidiabetic medications. CKD was defined as estimated glomerular filtration rate < 60 mL/min/1.73 m^2^, or persistent kidney abnormalities, such as proteinuria, ≥3-months [32]. Dominant hand, and history of hand surgery, were also evaluated in medical records. Regarding medications, the use of nonsteroidal anti-inflammatory drugs, disease-modifying antirheumatic drugs, prednisolone, methotrexate, bisphosphonates, vitamin D supplements, and other anti-osteoporosis drugs was investigated. For patients taking prednisolone or methotrexate, the dosage was recorded. The use of biological agents (infliximab, etanercept, adalimumab, tocilizumab, abatacept, golimumab, and certolizumab pegol) was also evaluated. Additionally, Steinbrocker’s staging and functional classification were assessed from medical records [33].

### 2.3. Radiological Examinations

**Figure 2:**
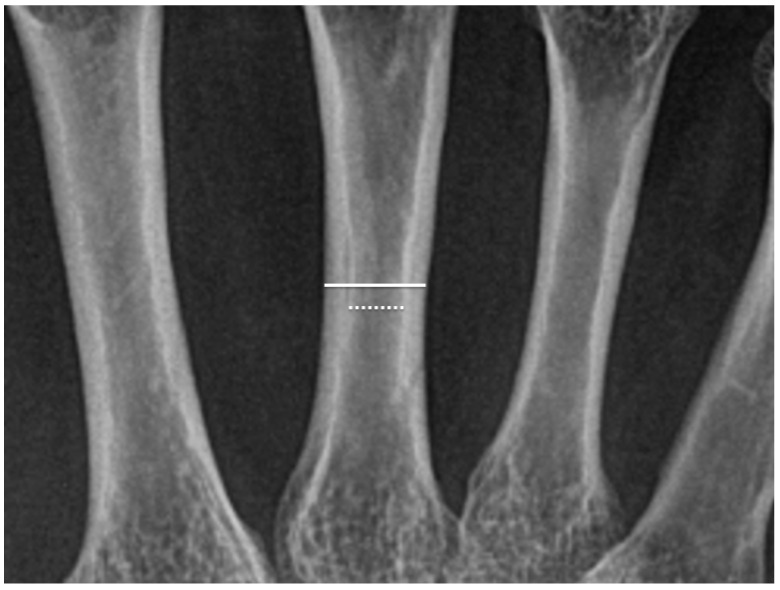
In this radiographic image of the metacarpal bone, the solid white line (upper line in the figure) represents the periosteal (outer) diameter, and the dotted white line (lower line in the figure) represents the endosteal (inner) diameter. These measurements are taken at the midpoint of the diaphysis. Cortical thickness is calculated as the difference between the outer and inner diameters, and the cortical thickness rate (CTR) is calculated by dividing the cortical thickness by the outer diameter.

X-rays of both hands were obtained using Fuji computed radiography (FCR 9000C, Fujifilm Corporation, Tokyo, Japan). Radiographs were examined in the electronic medical record system by one of two blinded readers. The method for evaluating cortical thickness rate (CTR) was based on the techniques described by Roldan et al. [30] and Haara et al. [25]. The outer (periosteal) diameter and inner (endosteal) diameter were measured at the midpoint of the diaphysis in the 2nd to 4th metacarpal bones of both hands. The CTR for each metacarpal was calculated as the thickness of the cortical bone (difference between outer and inner diameters) divided by the outer diameter (Figure 2). The average CTR across the 2nd to 4th metacarpals was then calculated and defined as the CTR for this study. Based on the report by Vehmas et al. [34], CTR < 0.50 was defined as low. CTR was evaluated in both hands and the metacarpal with lower CTR was designated “thin-side” while that with higher was designated “thick-side.” The ratio of thin- to thick-side CTRs was termed CTRR. In the absence of studies on CTRR laterality, from osteoporosis diagnostic criteria [35], we defined CTRR < 0.8 as CTRR lateral (+). Bone damage was classified as Stage II per Steinbrocker’s criteria [33]. Wrist joint damage (WJD), including damage of the wrist, carpal bones, and metacarpophalangeal (MP) joints of the 2nd to 4th fingers, was assessed in both hands. For WJD, patients with findings on only one side were classified as WJD lateral (+) and on both or neither side as WJD lateral (−).

The formula for CTR is as follows:

CTR = (outer diameter − inner diameter)/outer diameter

### 2.4. Statistical Analyses

Results are expressed as mean ± standard deviation for normally distributed data, while non-normally distributed data are presented as median with interquartile ranges (25th percentile [Q1] to 75th percentile [Q3]). For univariate analyses, differences in means between two groups were evaluated using the Mann–Whitney U test for continuous variables. For categorical data, Chi-square or Fisher’s exact test was applied. For nonparametric paired data, the Wilcoxon test was used. To evaluate correlations between continuous parametric data, Pearson’s correlation test was used. Multivariate analysis was performed using analysis of covariance (ANCOVA). Explanatory variables included those with significant correlations in univariate analysis as well as clinically relevant variables. Variables that did not have a normal distribution were logit-transformed to meet the normality assumption required for statistical analysis. SPSS version 26.0 was used for all statistical analyses with two-tailed *p* < 0.05 deemed statistically significant.

## 3. Results

### 3.1. Study Participants

This study included 143 RA patients (107 females, 36 males, mean ± SD: age 62.4 ± 12.1 yrs, disease duration 11.1 ± 9.3 yrs.) Demographic data are shown in Table 1. Smoking was reported in 25 patients (17.5%), alcohol consumption in 23 (16.1%), and 126 (88.1%) were right-handed. A history of hand surgery was reported in seven (4.9%), diabetes mellitus in eighteen (12.6%), and chronic kidney disease in six (4.2%) patients. As most study participants were outpatients, disease activity indices indicated remission or low activity (Table 1). Regarding medication, 4.8 ± 4.1 mg/week methotrexate was administered to 95 (66.4%), 1.8 ± 2.3 mg/day prednisolone to 73 (51.0%) and disease-modifying antirheumatic drugs to 47 patients (32.8%). Biologic agents were administered to 56 patients (39.2%). Bisphosphonates were used by forty-three (30.1%), vitamin D supplements by twenty-five (17.3%), and other anti-osteoporosis drugs by three patients (2.1%) (Table 1).

### 3.2. Metacarpal CTR

The mean bilateral CTR of the 2nd to 4th metacarpals was 0.423 ± 0.123, with the thin-side being 0.399 ± 0.125 and the thick-side 0.446 ± 0.124, making CTRR 0.890 ± 0.088. CTR < 0.500 was observed in 114 patients (79.7%) on the thin-side and 95 (66.4%) on the thick-side. In total, 19 patients (13.2%) showed CTR < 0.500 exclusively on the thin-side and 29 (20.3%) ≥ 0.500 on both sides (Table 2).

### 3.3. Damage to Wrist and Metacarpophalangeal Joints

Regarding WJD, 77 patients (53.8%) showed none, 24 (16.8%) showed unilateral and 42 (29.4%) bilateral damage. For MP joints, the figures were as follows: 2nd MP joint—one hundred and thirteen (79.0%), eight (5.6%), and twenty-two (15.4%) patients; 3rd MP joint—one hundred and seventeen (81.8%), twelve (8.4%), and fourteen (9.8%); and 4th MP joint—one hundred and twenty-two (85.9%), twelve (8.5%), and eight patients (5.6%), respectively (Table 2).

### 3.4. Unilateral Reduction of Metacarpal CTR

**Figure 3:**
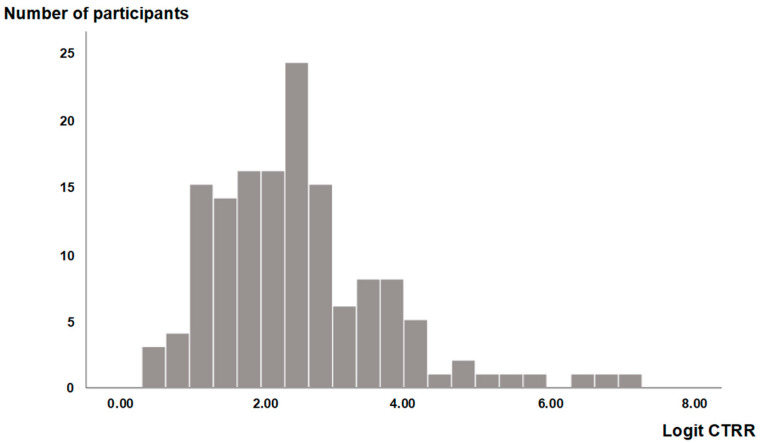
Histogram of Logit CTRR values. This histogram shows the distribution of Logit CTRR values. The *x*-axis represents Logit CTRR values, ranging from approximately 0 to 8, and the *y*-axis indicates the frequency of occurrences in each range. The distribution of this histogram closely approximates a normal distribution.

CTRR lateral (+) was observed in 24 patients (16.8%). Among the 20 patients with known hand dominance, 10 showed reduced cortical thickness on the dominant side, and 10 on the non-dominant side. A total of 119 patients (83.2%) had CTRR ≥ 0.8, classified as CTRR lateral (−). CTRR showed a non-normal distribution; therefore, a logit transformation was applied to approximate normality. This approximation was confirmed by examining the histogram of the transformed data (Figure 3).

### 3.5. Comparative Analysis of Laterality in Metacarpal CTR and WJD

In patients with WJD, a Chi-square test revealed a significant association between WJD and metacarpal CTR laterality where 50.0% of patients classified as WJD lateral (+) were also CTRR lateral (+), compared to only 10.1% of those classified as WJD lateral (−). Thus, unilateral WJD was associated with an almost 5-fold higher frequency of laterality in metacarpal cortical thickness. While this strong association was observed between CTR and WJD laterality (*p* < 0.01, Table 3), no significant association was found between WJD laterality and hand dominance, nor between WJD laterality and other parameters. In additional analysis, we compared 102 patients diagnosed with the 1987 criteria and later meeting the 2010 criteria (Old group), with 41 patients meeting the 2010 criteria from the start (New group). CTR lateral (+) was observed in twenty of the Old group and four of the New group with no significant difference between these groups (Chi-square test, *p* = 0.24). These findings suggest that the diagnostic criteria did not influence CTRR laterality (Supplementary Data, Table S1).

### 3.6. Relationship Between Thin- and Thick-Side CTRs, Logit CTRR, and Various Parameters (ANCOVA)

ANCOVA were conducted using thin- and thick-side CTRs as dependent variables to assess the effects of wrist joint lesions, sex (female), handedness, age, and interactions between thin- and thick-side WJDs. No significant interaction between thin- and thick-side WJDs was observed. Age was significant in both analyses (*p* < 0.001), whereas high *p*-values for sex and handedness indicated adjustment was unnecessary (Analysis 1, Table 4). ANCOVA was repeated with thin- and thick-side CTRs as dependent variables adjusted for age, excluding sex and handedness as parameters. Consistently, no significant interaction effect between thin- and thick-side WJDs was observed (Analysis 2, Table 4). Additional analyses were conducted excluding the interaction between thin- and thick-side WJDs. For thin-side CTR, significant effects were observed for thin-side WJD (*p* = 0.004), thick-side WJD (*p* = 0.005), and age (*p* < 0.001). For thick-side CTR, significant effects were found for thick-side WJD (*p* < 0.001) and age (*p* < 0.001), while no significant effect for thin-side WJD was observed (*p* = 0.627) (Analysis 3, Table 4). Further multiple comparisons were performed. For thin-side CTR, a significant difference was observed based on presence or absence of thin-side WJD (main effect test: *p* = 0.005, estimated marginal mean CTR difference of 0.05637) and thick-side WJD (main effect test: *p* = 0.004, estimated marginal mean CTR difference of 0.06035) (Table 5). For thick-side CTR, no significant difference was found based on presence or absence of thin-side WJD (main effect test: *p* = 0.627, mean CTR difference of 0.00956); however, a significant difference was observed for presence or absence of thick-side WJD (main effect test: *p* < 0.001, estimated marginal mean CTR difference of 0.093) (Table 5). ANCOVA was conducted with Logit CTRR as the dependent variable. Following the approach used in Analysis 2 for thin- and thick-side CTRs, adjustments were made for age, and the effects of thin-side WJD, thick-side WJD, and their interaction were examined. Neither age, thin-side WJD, nor thick-side WJD showed statistical significance, whereas interaction between thin- and thick-side WJDs was significant (Table 4). Further multiple comparisons were performed. In the simple main effects test, when thick-side WJD was absent, Logit CTRR was significantly lower in the presence of thin-side WJD compared to its absence (mean difference: −1.461, OR: 0.232, *p* < 0.001). When thin-side WJD was present, Logit CTRR was significantly higher in the presence of thick-side WJD compared to its absence (mean difference: 0.833, OR: 2.300, *p* = 0.014). In contrast, no significant difference in Logit CTRR was observed between the presence and absence of thin-side WJD when thick-side WJD was present or between presence and absence of thick-side WJD when thin-side WJD was absent (Table 5). Additional analyses revealed that neither rheumatoid factor (RF) and/or anti-CCP Ab nor prednisolone use had a significant impact on thin-CTR, thick-CTR or CTRR (Supplementary Data, Tables S2 and S3). Prednisolone use had also no significant impact on the laterality of joint damage (Supplementary Data, Table S4).

### 3.7. CTR Evaluation in Right and Left Hands Pre- and Post-Biologic Therapy in 30 Patients

In 30 patients, CTR was evaluated in both hands pre- and post-biologic therapy. CTR significantly decreased from 0.460 ± 0.105 to 0.413 ± 0.107 in the right hand and from 0.457 ± 0.106 to 0.423 ± 0.113 in the left hand (Table 6). Among patients with CTR ≥ 0.50 at baseline, significant reductions were also observed: right hand from 0.569 ± 0.275 to 0.497 ± 0.0855 and left hand from 0.567 ± 0.390 to 0.506 ± 0.102 (Table 6). Analysis of 30 cases revealed that the duration of biologic agent administration had no significant effect on CTRs (Supplementary Data, Table S5), and differences in effects among agents could not be evaluated due to small sample sizes.

## 4. Discussion

Metacarpal bone density decrease was bilateral in 66.4% and relatively unilateral in 16.8% of the RA patients studied. The latter was closely associated with ipsilateral WJD, suggesting asymmetrical WJD is involved in unilateral decreased metacarpal bone density.

Roldan et al. [30] reported that cortical bone loss in the metacarpals of RA patients correlated with the length of the observation period, identifying erythrocyte sedimentation rate and total steroid dosage as independent factors in multivariate analysis. This demonstrated an association between systemic inflammation and decreased metacarpal bone density. In our study, Logit CTRR was analyzed as the dependent variable using ANCOVA, adjusted for age and including thin-side WJD, thick-side WJD, and their interaction. Age, thin-side WJD, and thick-side WJD were not significant factors, whereas the interaction was. Further multiple comparisons showed that under conditions without thick-side WJD, Logit CTRR was significantly lower when thin-side WJD was present than when absent (mean difference: −1.461, OR: 0.232, *p* < 0.001). With thin-side WJD present, Logit CTRR was significantly higher when thick-side WJD was also present (mean difference: 0.833, OR: 2.300, *p* = 0.014). The lowest Logit CTRR, indicating the greatest laterality in CTR, was observed when thick-side WJD was absent and thin-side WJD was present. This suggests unilateral WJD is reflected in the laterality of CTR. Previous research has shown that inflammatory bone loss in periarticular osteoporosis of the hand affects not only periarticular regions but the cortical bone of the metacarpal diaphysis, independent of direct synovial contact [15,17,18]. Our findings suggest inflammation in the hand joints, likely through localized cytokine release, contributed to reduction in cortical bone density of the ipsilateral metacarpal diaphysis.

RA is generally characterized by symmetrical joint involvement [36], but asymmetrical arthritis has been reported in some patients [28,29,37]. Halla et al. [37] examined small joints of the hands and feet and found asymmetrical involvement was common, with 90% of wrist joints showing symmetrical involvement and 10% asymmetry. Similarly, Zangger et al. [29] found asymmetrical joint involvement in small joints of the hands and feet in 13–16% of cases. In our study, unilateral WJD was observed in 24 patients (16.8%), consistent with previous reports, suggesting that localized factors such as cytokine release may contribute to asymmetrical metacarpal bone density reduction. Koh JH [38] and Yaku A [39] reported faster bone destruction progression in the dominant hand. However, in our study, we observed no significant difference between right and left sides nor any effect by hand dominance. This may be due to the use of different radiographic methods or that only six of the one hundred and thirty-two patients with hand dominance recorded were left-handed, preventing reliable statistical analysis of handedness. The potential contribution of repetitive manual tasks to localized joint stress and asymmetry has been reported previously [40]. Although we did not collect detailed occupational data in our cohort, this hypothesis may provide a possible explanation for the risk of asymmetric joint damage and bone density reduction. Genetic predispositions—such as the involvement of HLA-DR4 in rheumatoid arthritis [41] and WNT16 in cortical bone thickness [42]—may contribute to asymmetric joint damage and bone density reduction. However, genetic marker data were not collected in this study, representing a limitation for future investigation.

CTR values for both right and left hands were evaluated pre- and post-administration of biologic agents in 30 patients. CTR was significantly decreased post-treatment even in patients with baseline CTR ≥ 50 (Table 6). Hoff et al. [43] evaluated the effect of adalimumab on metacarpal bone loss, finding a significant reduction in bone loss rate in their adalimumab + MTX treatment group compared to MTX monotherapy, though without improvement in bone density. Bone loss rates of 1.15%, 2.16%, and 3.03% were observed at 26, 52, and 104 weeks, respectively. Their results match our finding of no improvement in metacarpal bone density by biologic treatment, suggesting that systemic effects beyond local joint inflammation were not fully suppressed, leading to continued CTR decrease. CTR evaluation should consider both local and systemic effects. Aggressive RA treatment with biologic agents may be necessary to suppress cytokine release from wrist joints before CTR decline begins. Additionally, Baker et al. [44] reported that muscle loss contributes to cortical bone density reduction, suggesting that rehabilitation interventions could also be beneficial. The early detection of unilateral bone erosions using MRI, followed by intensive treatment, may offer a viable strategy for managing asymmetric joint damage. While biological agents were ineffective in preventing cortical bone loss in this study, the potential of JAK inhibitors to suppress early arthritis and mitigate asymmetric joint damage deserves further exploration in future studies.

This study has several limitations. First, it involved a relatively small sample size from two institutions. Second, as the study focused on outpatients with relatively stable disease, the findings may not apply to all RA patients in terms of disease control. These factors may limit the generalizability of the results. Third, the single time-point analysis prevented assessment of longitudinal change in cortical bone or wrist joint damage. Fourth, image evaluation was performed manually using Fuji computed radiography, rather than computer-assisted digital X-ray radiogram (DXR), which may have introduced observer bias. Fifth, the lack of detailed occupational data may have limited a deeper understanding of the potential influence of repetitive manual tasks on joint stress and asymmetry. Future studies should include larger more diverse populations, use technologies such as DXR, and include longitudinal assessments to strengthen the findings.

## 5. Conclusions

This study is the first to report that a substantial number of RA patients exhibited decreased metacarpal bone density, with a subset displaying unilateral bone loss. Additionally, the relationship between unilateral bone loss and wrist joint damage was identified for the first time. Wrist joint inflammation was found to be associated with cortical bone loss in the metacarpals, suggesting that local cytokine release contributed to this reduction. Despite biologic treatment, no improvement in metacarpal bone loss was observed. Although previous reports have discussed reduced metacarpal bone density in RA patients, no detailed studies had focused on laterality or asymmetry until this report.

## Figures and Tables

**Table 1:** Characteristics of study participants.

Variable	Values (*n* = 143)
Age (yrs.)	Median (IQR): 64 (54–71)
Sex (female/male)	107/36
Disease duration (yrs)	Median (IQR): 8 (4–14)
Height (cm)	156.9 ± 9.4
Weight (kg)	Median (IQR): 52.3 (46.2–61.4)
Body mass index	22.0 ± 3.1
Smoking, n (%)	25 (17.5%)
Alcohol drinking, n (%)	23 (16.1%)
Right-handedness, n (%)	126 (88.1%)
Operation (hand), n (%)	7 (4.9%)
Diabetes mellitus, n (%)	18 (12.6%)
Chronic kidney disease, n (%)	6 (4.2%)
CRP (mg/dL)	Median (IQR): 0.10 (0.00–0.30)
ESR (mm/1 h)	Median (IQR): 16 (9–29)
DAS28-CRP	Median (IQR): 1.73 (1.24–2.40)
DAS28-ESR	2.79 ± 1.10
CDAI	Median (IQR): 2.2 (0–5.16)
SDAI	Median (IQR): 2.4 (0.3–5.5)
RF positive, n (%)	92/113 (81.4%)
ACPA positive, n (%)	53/71 (74.6%)
Methotrexate, n (%)	95 (66.4%)
Dose (mg/week)	4.8 ± 4.1
Prednisolone, n (%)	73 (51.0%)
Dose (mg/day)	1.8 ± 2.5
DMARDs, n (%)	47 (32.8%)
Biologic therapy, n (%)	56 (39.2%)
Bisphosphonate, n (%)	43 (30.1%)
Vitamin D, n (%)	25 (17.5%)
Other anti-osteoporosis drug, n (%)	3 (2.1%)

Median (Q1–Q3): The median value is presented with the interquartile range (25th percentile [Q1] to 75th percentile [Q3]). Mean ± SD: The mean value is presented with its standard deviation (SD). Age (yrs.): Age in years. Sex (female/male): Number of female and male participants. Disease duration (yrs.): Duration of disease in years. Height (cm): Participant height in centimeters. Weight (kg): Participant weight in kilograms. Body mass index: Body mass index calculated as weight (kg) divided by height squared (m^2^). Smoking, n (%): Number and percentage of participants who smoke. Alcohol drinking, n (%): Number and percentage of participants who consume alcohol. Right-handedness, n (%): Number and percentage of participants with right-handed dominance. Operation (hand), n (%): Number and percentage of participants who underwent hand surgery. Diabetes mellitus, n (%): Number and percentage of participants with diabetes. Chronic kidney disease, n (%): Number and percentage of participants with chronic kidney disease. CRP (mg/dL): C-reactive protein levels in milligrams per deciliter. ESR (mm/1 h): Erythrocyte sedimentation rate in millimeters per hour. DAS28-CRP: Disease Activity Score in 28 joints with CRP. DAS28-ESR: Disease Activity Score in 28 joints with Erythrocyte Sedimentation Rate. CDAI: Clinical Disease Activity Index. SDAI: Simplified Disease Activity Index. RF positive, n (%): Number and percentage of participants testing positive for rheumatoid factor. ACPA positive, n (%): Number and percentage of participants testing positive for anti-citrullinated protein antibody. Methotrexate, n (%): Number and percentage of participants treated with methotrexate. Dose (mg/week): Weekly dose of methotrexate in milligrams. Prednisolone, n (%): Number and percentage of participants treated with prednisolone. Dose (mg/day): Daily dose of prednisolone in milligrams. DMARDs, n (%): Number and percentage of participants treated with disease-modifying antirheumatic drugs (DMARDs). Biologic therapy, n (%): Number and percentage of participants receiving biologic therapies. Bisphosphonate, n (%): Number and percentage of participants treated with bisphosphonates f. Vitamin D, n (%): Number and percentage of participants treated with vitamin D. Other anti-osteoporosis drug, n (%): Number and percentage of participants treated with non-bisphosphonate anti-osteoporosis drugs.

**Table 2:** Characteristics of study participants.

	Values (*n* = 143)
Thin-side CTR	0.399 ± 0.125
Thick-side CTR	0.446 ± 0.124
Mean of thin- and thick-side CTRs	0.423 ± 0.123
Low CTR (thin-side), n (%)	114 (79.7%)
Low CTR (thick-side), n (%)	95 (66.4%)
Low CTR (only thin-side), n (%)	19 (13.2%)
Normal CTR (both thin- and thick-sides), n (%)	29 (20.3%)
Joint damage	
Wrist joint: none, n (%)	77 (53.8%)
Wrist joint: unilateral, n (%)	24 (16.7%)
Wrist joint: bilateral, n (%)	42 (29.4%)
2nd MP joint: none, n (%)	113 (79.0%)
2nd MP joint: unilateral, n (%)	8 (5.6%)
2nd MP joint: bilateral, n (%)	22 (15.4%)
3rd MP joint: none, n (%)	117 (81.8%)
3rd MP joint: unilateral, n (%)	12 (8.4%)
3rd MP joint: bilateral, n (%)	14 (9.8)
4th MP joint: none, n (%)	122 (85.3%)
4th MP joint: unilateral, n (%)	12 (8.4%)
4th MP joint: bilateral, n (%)	8 (5.6%)

Mean ± SD: The mean value is presented with its standard deviation (SD). CTR: cortical thickness rate. In each patient, the side with the lower cortical thickness rate (CTR) of the metacarpals was designated the “thin-side” and that with the higher CTR the “thick-side”. Low CTR (thin-side), n (%): proportion of cases with CTR < 0.50 on the thin-side. Low CTR (thick-side), n (%): proportion of cases with CTR < 0.50 on the thick-side. Low CTR (only thin-side), n (%): proportion of cases with CTR < 0.50 on thin-side only. Normal CTR (both thin- and thick-side), n (%): proportion of cases with CTR > 0.50 on both sides. Wrist joint/2nd–4th MP joint: none, n (%): No lesions detected. Wrist joint/2nd–4th MP joint: unilateral, n (%): Lesions present on one side (right or left). Wrist joint/2nd–4th MP joint: bilateral, n (%): Lesions present on both sides (right and left). MP joint: Metacarpophalangeal joint.

**Table 3:** Comparison of laterality in wrist joint damage and cortical thickness rate ratio (CTRR) in RA Patients.

	WJD Lateral (+)	WJD Lateral (−)	
CTRR lateral (+)	12	12	24
CTRR lateral (−)	12	107	119
	24	119	143

CTRR: cortical thickness rate ratio = CTR (thin-side)/CTR (thick-side). CTRR lateral (+) = CTRR < 0.8. CTRR lateral (−) = CTRR ≥ 0.8. WJD: wrist joint damage. WJD lateral (+) = WJD in one hand. WJD lateral (−) = WJD in both hands or no hand. WJD lateral (+) and CTRR lateral (+) were significantly related: χ^2^ test (*p* < 0.001).

**Table 4:** Analysis of covariance between thin-side CTR, thick-side CTR, Logit CTRR, and associated parameters.

		Analysis 1			Analysis 2			Analysis 3		
		F	df	*p*-value	F	df	*p*-value	F	df	*p*-value
Thin-side CTR	Age	63.54	1, 125	<0.001 **	72.18	1, 138	<0.001 **	72.78	1, 139	<0.001 **
	Sex (Female)	0.99	1, 125	0.32	not included			not included		
	Dominant hand	0.00	1, 125	0.95	not included			not included		
	Thin-side WJD	334.40	1, 8.041	<0.001 **	33.60	1, 1.060	0.10	8.00	1, 136	0.005 **
	Thick-side WJD	114.22	1, 8.041	<0.001 **	46.58	1, 1.030	0.09	8.69	1, 139	0.004 **
	Interaction ^†^	0.01	1, 125	0.92	0.19	1, 138	0.67	not included		
		Analysis 1			Analysis 2			Analysis 3		
		F	df	*p*-value	F	df	*p*-value	F	df	*p*-value
Thick-side CTR	Age	72.78	1, 125	<0.001 **	86.36	1, 138	<0.001 **	86.22	1, 139	<0.001 **
	Sex (Female)	0.91	1, 125	0.34	not included			not included		
	Dominant hand	0.00	1, 125	0.96	not included			not included		
	Thin-side WJD	1.83	1, 1.028	0.40	0.72	1, 1.014	0.55	0.24	1, 139	0.63
	Thick-side WJD	6.64	1, 1.023	0.23	23.74	1, 1.007	0.13	21.10	1, 139	<0.001 **
	Interaction ^†^	1.28	1, 125	0.26	0.77	1, 138	0.38	not included		
		Analysis 2								
		F	df	*p*-value						
Logit CTRR	Age	0.41	1, 138	0.52						
	Thin-side WJD	2.95	1, 1.003	0.34						
	Thick-side WJD	0.30	1, 1.001	0.68						
	Interaction ^†^	3.94	1, 138	<0.05 *						

CTR: cortical thickness rate. In each patient, the side with the lower cortical thickness rate (CTR) of the metacarpals was designated the “thin-side” and that with the higher CTR the “thick-side”. WJD: wrist joint damage. CTRR: cortical thickness rate ratio = CTR (thin-side)/CTR (thick-side). Logit CTRR = ln {CTRR/(1-CTRR)}. To approximate a normal distribution for analysis of covariance (ANCOVA), CTRR was transformed using logit transformation. This involved applying the logit function to CTRR values, defined as: Logit CTRR = ln {CTRR/(1-CTRR)}. Interaction ^†^: interaction between thin- and thick-side WJDs. Analysis 1: ANCOVA with the following dependent variable and factors: age, sex (female), dominant hand, WJD thin-side, WJD thick-side, and interaction between thin- and thick-side WJDs. Analysis 2: ANCOVA with the following dependent variable and factors: age, WJD thin-side, WJD thick-side, and interaction between thin- and thick-side WJDs. (excluding sex (female) and dominant hand in Analysis 1). Analysis 3: ANCOVA with the following dependent variable and factors: age, WJD thin-side, WJD thick-side (excluding interaction between thin- and thick-side WJDs in Analysis 2). F: F-value. df: degrees of freedom, expressed as F (df1, df2), where df1 is df for the factor between-groups and df2 is df for the error within-groups. *: significant, *p*-value < 0.05. **: significant, *p*-value < 0.01.

**Table 5:** Effect of WJD on thin-side CTR, thick-side CTR, and CTRR: post-hoc analysis of ANCOVA.

	Thinner side CTR, Analysis 3					
	Thick-side WJD (−)	Thick-side WJD (+)	EM mean	Main effect	*p*-value	
Thin-side WJD (−)	0.443	0.383	0.413	−0.056	0.005 **	
Thin-side WJD (+)	0.387	0.326	0.356			
EM mean	0.415	0.354				
Main effect	−0.060					
*p*-value	0.004 **					
	Thicker side CTR, Analysis 3					
	Thick-side WJD (−)	Thick-side WJD (+)	EM mean	Main effect	*p*-value	
Thin-side WJD (−)	0.482	0.390	0.436	−0.010	0.63	
Thin-side WJD (+)	0.473	0.380	0.426			
EM mean	0.477	0.385				
Main effect	−0.093					
*p*-value	<0.001 **					
	Logit CTRR, Analysis 2					
	Thick-side WJD (−)	Thick-side WJD (+)		Simple main effect	OR	*p*-value
Thin-side WJD (−)	2.883 a	2.642 b	b-a	−0.242	0.785	0.57
Thin-side WJD(+)	1.423 c	2.255 d	d-c	0.833	2.300	0.014 *
	c-a	d-b				
Simple main effect	−1.461	−0.386				
OR	0.232	0.680				
*p*-value	<0.001 **	0.38				

In each patient, the side with the lower cortical thickness rate (CTR) of the metacarpals was designated the “thin-side” and that with the higher CTR the “thick-side”. WJD: wrist joint damage. CTRR: cortical thickness rate ratio = CTR (thin-side)/CTR (thick-side). Logit CTRR = ln {CTRR/(1-CTRR)}. To ensure normality of variables for the analysis of covariance (ANCOVA), the variable CTRR was transformed using a logit transformation. This process involved applying the logit function to CTRR values, defined as: Logit CTRR = ln {CTRR/(1-CTRR)}. Analysis 1: ANCOVA with the following dependent variable and factors: age, sex (female), dominant hand, WJD thin-side, WJD thick-side, and interaction between thin- and thick-side WJDs. Analysis 2: ANCOVA with the following dependent variable and factors: age, WJD thin-side, WJD thick-side, and interaction between thin- and thick-side WJDs. (excluding sex (female) and dominant hand in Analysis 1). Analysis 3: ANCOVA with the following dependent variable and factors: age, WJD thin-side, WJD thick-side (excluding interaction between thin- and thick-side WJDs in Analysis 2). EM = estimated marginal mean, the adjusted mean accounting for covariates in the ANCOVA model. OD: odds ratio. *: significant, *p*-value < 0.05. **: significant, *p*-value < 0.01.

**Table 6:** CTR of right and left hands pre- and post-biologic therapy.

	Pre-Biologics	Post-Biologics	Number	*p*-Value
CTR (right)	0.460 ± 0.105	0.413 ± 0.107	30	<0.001 **
CTR (left)	0.457 ± 0.106	0.423 ± 0.113	30	0.029 *
CTR (right) #	0.569 ± 0.275	0.497 ± 0.855	11	0.018 *
CTR (left) #	0.567 ± 0.390	0.506 ± 0.102	10	0.041 *

#: CTR ≥ 0.50 at baseline. *: significant, *p*-value < 0.05. **: significant, *p*-value < 0.01.

## Data Availability

The data presented in this study are available upon reasonable request from the corresponding author. Due to privacy restrictions, data containing patient information are not publicly available.

## Supplementary Materials

The following supporting information can be downloaded at: https://www.mdpi.com/article/10.3390/jcm13247652/s1, Table S1: Comparison of CTRR Laterality (+/−) and Grouping by Old/New Classification in RA Patients; Table S2: Analysis of covariance between thin-side CTR, thick-side CTR, Logit CTRR and associated parameters including RF and/or CCP; Table S3: Analysis of covariance between Logit CTRR and associated parameters including prednisolone; Table S4: Comparison of Laterality in Wrist Joint Damage and Prednisolone administration in RA Patients; Table S5: Analysis of covariance between differences of thin-side CTR and thick-side CTR and duration of treatment with biologic agents.

## References

[1] Gravallese E.M., Firestein G.S. (2023). Rheumatoid arthritis—Common origins, divergent mechanisms. N. Engl. J. Med..

[2] Gough A.K., Lilley J., Eyre S., Holder R.L., Emery P. (1994). Generalised bone loss in patients with early rheumatoid arthritis. Lancet.

[3] Kroot E.J., Nieuwenhuizen M.G., de Waal Malefijt M.C., van Riel P.L., Pasker-de Jong P.C., Laan R.F. (2001). Change in bone mineral density in patients with rheumatoid arthritis during the first decade of the disease. Arthritis Rheum..

[4] Laan R.F., Buijs W.C., Verbeek A.L., Draad M.P., Corstens F.H., van de Putte L.B., van Riel P.L. (1993). Bone mineral density in patients with recent onset rheumatoid arthritis: Influence of disease activity and functional capacity. Ann. Rheum. Dis..

[5] Chan E., Pandith V., Towheed T.E., Brouillard D., Zee B., Anastassiades T.P. (1998). Comparison of the combined cortical thickness of the second metacarpal with Sharp’s method for scoring hand microradiographs in rheumatoid arthritis. J. Rheumatol..

[6] Bayraktar H.H., Morgan E.F., Niebur G.L., Morris G.E., Wong E.K., Keaveny T.M. (2004). Comparison of the elastic and yield properties of human femoral trabecular and cortical bone tissue. J. Biomech..

[7] Aguado F., Revilla M., Villa L.F., Rico H. (1997). Cortical bone resorption in osteoporosis. Calcif. Tissue Int..

[8] Böttcher J., Pfeil A., Heinrich B., Lehmann G., Petrovitch A., Hansch A., Heyne J., Mentzel H., Malich A., Hein G. (2005). Digital radiogrammetry as a new diagnostic tool for estimation of disease-related osteoporosis in rheumatoid arthritis compared with pQCT. Rheumatol. Int..

[9] Bottcher J., Pfeil A. (2008). Diagnosis of periarticular osteoporosis in rheumatoid arthritis using digital X-ray radiogrammetry. Arthritis Res. Ther..

[10] Alenfeld F.E., Diessel E., Brezger M., Sieper J., Felsenberg D., Braun J. (2000). Detailed analyses of periarticular osteoporosis in rheumatoid arthritis. Osteoporos. Int..

[11] Deodhar A.A., Brabyn J., Jones P.W., Davis M.J., Woolf A.D. (1995). Longitudinal study of hand bone densitometry in rheumatoid arthritis. Arthritis Rheum..

[12] Sambrook P.N. (2000). The skeleton in rheumatoid arthritis: Common mechanisms for bone erosion and osteoporosis. J. Rheumatol..

[13] Dolan A.L., Moniz C., Abraha H., Pitt P. (2002). Does active treatment of rheumatoid arthritis limit disease-associated bone loss. Rheumatology.

[14] Wolfe F., Sharp J.T. (1998). Radiographic outcome of recent-onset rheumatoid arthritis: A 19-year study of radiographic progression. Arthritis Rheum..

[15] Lems W.F. (2007). Bisphosphonates and glucocorticoids: Effects on bone quality. Arthritis Rheum..

[16] Kong Y.Y., Feige U., Sarosi I., Bolon B., Tafuri A., Morony S., Capparelli C., Li J., Elliott R., McCabe S. (1999). Activated T cells regulate bone loss and joint destruction in adjuvant arthritis through osteoprotegerin ligand. Nature.

[17] Haugeberg G., Ørstavik R.E., Kvien T.K. (2003). Effects of rheumatoid arthritis on bone. Curr. Opin. Rheumatol..

[18] Güler-Yüksel M., Allaart C.F., Goekoop-Ruiterman Y.P.M., de Vries-Bouwstra J.K., van Groenendael J.H.L.M., Mallée C., de Bois M.H.W., Breedveld F.C., Dijkmans B.A.C., Lems W.F. (2009). Changes in hand and generalised bone mineral density in patients with recent-onset rheumatoid arthritis. Ann. Rheum. Dis..

[19] Kalla A.A., Meyers O.L., Chalton D., Heath S., Brown G.M.M., Smith P.R., Burger M.C. (1991). Increased metacarpal bone mass following 18 months of slow-acting antirheumatic drugs for rheumatoid arthritis. Br. J. Rheumatol..

[20] Jensen T., Hansen M., Jensen K.E., Podenphant J., Hansen T.M., Hyldstrup L. (2005). Comparison of dual X-ray absorptiometry (DXA), digital X-ray radiogrammetry (DXR), and conventional radiographs in the evaluation of osteoporosis and bone erosions in patients with rheumatoid arthritis. Scand. J. Rheumatol..

[21] Pfeil A., Haugeberg G., Renz D.M., Reinhardt L., Jung C., Franz M., Wolf G., Böttcher J. (2017). Digital X-ray radiogrammetry and its sensitivity and specificity for the identification of rheumatoid arthritis-related cortical hand bone loss. J. Bone Min. Metab..

[22] Hoff M., Haugeberg G., Kvien T.K. (2007). Hand bone loss as an outcome measure in established rheumatoid arthritis: 2-year observational study comparing cortical and total bone loss. Arthritis Res. Ther..

[23] Book C., Algulin J., Nilsson J.A., Saxne T., Jacobsson L. (2009). Bone mineral density in the hand as a predictor for mortality in patients with rheumatoid arthritis. Rheumatology.

[24] Wilczek M.L., Kälvesten J., Algulin J., Beiki O., Brismar T.B. (2013). Digital X-ray radiogrammetry of hand or wrist radiographs can predict hip fracture risk—A study in 5,420 women and 2,837 men. Eur. Radiol..

[25] Haara M., Heliövaara M., Impivaara O., A Arokoski J.P., Manninen P., Knekt P., Kärkkäinen A., Reunanen A., Aromaa A., Kröger H. (2006). Low metacarpal index predicts hip fracture: A prospective population study of 3,561 subjects with 15 years of follow-up. Acta Orthop..

[26] Towheed T.E., Brouillard D., Yendt E., Anastassiades T. (1995). Osteoporosis in rheumatoid arthritis: Findings in the metacarpal, spine, and hip and a study of the determinants of both localized and generalized osteopenia. J. Rheumatol..

[27] Arnett F.C., Edworthy S.M., Bloch D.A., Mcshane D.J., Fries J.F., Cooper N.S., Healey L.A., Kaplan S.R., Liang M.H., Luthra H.S. (1988). The American Rheumatism Association 1987 revised criteria for the classification of rheumatoid arthritis. Arthritis Rheum..

[28] Hamilton S. (1983). Unilateral rheumatoid arthritis in hemiplegia. J. Can. Assoc. Radiol..

[29] Zangger P., Keystone E.C., Bogoch E.R. (2005). Asymmetry of small joint involvement in rheumatoid arthritis: Prevalence and tendency towards symmetry over time. Jt. Bone Spine..

[30] Roldan J.F., Del Rincon I., Escalante A. (2006). Loss of cortical bone from the metacarpal diaphysis in patients with rheumatoid arthritis: Independent effects of systemic inflammation and glucocorticoids. J. Rheumatol..

[31] Aletaha D., Neogi T., Silman A.J., Funovits J., Felson D.T., Bingham C.O., Birnbaum N.S., Burmester G.R., Bykerk V.P., Cohen M.D. (2010). 2010 rheumatoid arthritis classification criteria: An American College of Rheumatology/European League Against Rheumatism collaborative initiative. Ann. Rheum. Dis..

[32] Levin A., Stevens P.E. (2014). Summary of KDIGO 2012 CKD Guideline: Behind the scenes, need for guidance, and a framework for moving forward. Kidney Int..

[33] Steinbrocker O., Traeger C.H., Batterman R.C. (1949). Therapeutic criteria in rheumatoid arthritis. J. Am. Med. Assoc..

[34] Vehmas T., Solovieva S., Riihimaki H., Luoma K., Leino-Arjas P. (2005). Hand workload and the metacarpal cortical index. a study of middle-aged teachers and dentists. Osteoporos. Int..

[35] Hagino H. (2014). Revised osteoporosis diagnostic criteria and Japanese practice guideline on osteoporosis. Clin. Calcium..

[36] Clarke G.S., Buckland-Wright J.C., Grahame R. (1994). Symmetry of radiological features in the wrist and hands of patients with early to moderate rheumatoid arthritis: A quantitative microfocal radiographic study. Br. J. Rheumatol..

[37] Halla J.T., Fallahi S., Hardin J.G. (1986). Small joint involvement: A systematic roentgenographic study in rheumatoid arthritis. Ann. Rheum. Dis..

[38] Koh J.H., Jung S.M., Lee J.J., Kang K.Y., Kwok S.-K., Park S.-H., Ju J.H. (2015). Radiographic structural damage is worse in the dominant than the non-dominant hand in individuals with early rheumatoid arthritis. PLoS ONE.

[39] Yaku A., Hashimoto M., Furu M., Ito H., Yamakawa N., Yamamoto W., Fujii T., Matsuda F., Mimori T., Terao C. (2016). Relationship between handedness and joint involvement in rheumatoid arthritis. Sci. Rep..

[40] Zakaria D., Robertson J., MacDermid J., Hartford K., Koval J. (2002). Work-related cumulative trauma disorders of the upper extremity: Navigating the epidemiologic literature. Am. J. Ind. Med..

[41] Miyadera H., Tokunaga K. (2015). Associations of human leukocyte antigens with autoimmune diseases: Challenges in identifying the mechanism. J. Hum. Genet..

[42] Zheng H.F., Tobias J.H., Duncan E., Evans D.M., Eriksson J., Paternoster L., Yerges-Armstrong L.M., Lehtimäki T., Bergström U., Kähönen M. (2012). WNT16 influences bone mineral density, cortical bone thickness, bone strength, and osteoporotic fracture risk. PLoS Genet..

[43] Hoff M., Kvien T.K., Kälvesten J., Elden A., Haugeberg G. (2009). Adalimumab therapy reduces hand bone loss in early rheumatoid arthritis: Explorative analyses from the PREMIER study. Ann. Rheum. Dis..

[44] Baker J.F., Long J., Mostoufi-Moab S., Denburg M., Jorgenson E., Sharma P., Zemel B.S., Taratuta E., Ibrahim S., Leonard M.B. (2017). Muscle Deficits in Rheumatoid Arthritis Contribute to Inferior Cortical Bone Structure and Trabecular Bone Mineral Density. J. Rheumatol..

