# Supplementary material for: Asymmetrical Damage of the Wrist Joint Induces Lateralized Cortical Bone Loss in the Metacarpal Diaphysis in Patients with Rheumatoid Arthritis
Source: J Clin Med. 2024 Dec 16;13(24):7652. doi: 10.3390/jcm13247652 (PMC11676186; doi:10.3390/jcm13247652)
Supplement: Supplementary file 1 [file jcm-13-07652-s001.zip › Supplementary Data/Table S1.pdf]

Table S1 Comparison of CTRR Laterality (+/-) and Grouping by Old/New Classification in RA Patients

	New Group	Old Group	
CTRR lateral (+)	4	20	24
CTRR lateral (-)	37	82	119
	41	102	143

CTRR: cortical thickness rate ratio=CTR (thin-side) / CTR (thick-side)

CTRR lateral (+) = CTRR < 0.8

CTRR lateral (-) = CTRR ≥ 0.8

New Group: Patients diagnosed based on the 2010 classification criteria from the beginning.

Old Group: Patients initially diagnosed based on the 1987 classification criteria and later meeting the 2010 classification criteria.

The relationship between classification group (Old/New) and CTRR laterality (+/-) was not statistically significant (χ^2 test, $p = 0.24$).
