# Supplementary material for: Asymmetrical Damage of the Wrist Joint Induces Lateralized Cortical Bone Loss in the Metacarpal Diaphysis in Patients with Rheumatoid Arthritis
Source: J Clin Med. 2024 Dec 16;13(24):7652. doi: 10.3390/jcm13247652 (PMC11676186; doi:10.3390/jcm13247652)
Supplement: Supplementary file 1 [file jcm-13-07652-s001.zip › Supplementary Data/Table S2.pdf]

Table S2. Analysis of covariance between thin-side CTR, thick-side CTR, Logit CTRR and associated parameters including RF and/or CCP

		Analysis 4				Analysis 5		
		F	df	p-value		F	df	p-value
Thin-side CTR	Age	61.454	1,106	<0.001		63.689	1, 109	<0.001
	RF and/or CCP	0.137	1, 1.873	0.75		0.398	1, 0.865	0.65
	Thin-side WJD	4.080	1, 1.018	0.29		11.896	1, 1.914	0.08
	Thick-side WJD	4.745	1, 1.021	0.27		6.719	1, 109	0.011
	Interaction†1	2.891	1, 0.400	0.53		not included		
	Interaction†2	50.309	1, 1.641	0.032		0.398	1, 109	0.53
	Interaction†3	121.798	1, 0.738	0.102		not included		
	Interaction†4	0.016	1, 106	0.90		not included		
		Analysis 4				Analysis 6		
		F	df	p-value		F	df	p-value
Thick-side CTR	Age	68.582	1, 106	<0.001		70.915	1, 110	<0.001
	RF and/or CCP	0.005	1, 0.426	0.97		0.020	1, 110	0.90
	Thin-side WJD	0.346	1, 0.309	0.77		1.190	1, 110	0.28
	Thick-side WJD	4.787	1,0.940	0.29		17.192	1, 110	<0.001
	Interaction†1	1.679	1, 0.991	0.42		not included		
	Interaction†2	0.440	1, 1.007	0.63		not included		
	Interaction†3	2.110	1, 0.996	0.38		not included		

	Interaction†4	1.273	1, 106	0.26		not included		
		Analysis 4				Analysis 6		
		F	df	p-value		F	df	p-value
Logit CTRR	Age	0.016	1, 106	0.90		0.091	1, 109	0.76
	RF and/or CCP	1.928	1, 0.031	0.92		1.217	1, 109	0.27
	Thin-side WJD	0.614	1, 0.936	0.58		3.270	1, 0.994	0.32
	Thick-side WJD	0.300	1, 0.427	0.75		0.168	1, 0.985	0.75
	Interaction†1	3.168	1, 0.992	0.33		3.492	1, 109	0.06
	Interaction†2	1.277	1, 1.006	0.46				
	Interaction†3	0.004	1, 0.997	0.96				
	Interaction†4	1.534	1, 106	0.22				

CTR: cortical thickness rate

In each patient, the side with the lower cortical thickness rate (CTR) of the metacarpals was designated the "thin-side" and that with the higher CTR the "thick-side."

WJD: wrist joint damage

CTRR: cortical thickness rate ratio= CTR (thin-side) / CTR (thick-side)

Logit CTRR=ln {CTRR/(1-CTRR)}

To approximate a normal distribution for analysis of covariance (ANCOVA), CTRR was transformed using logit transformation. This involved applying the logit function to CTRR values, defined as: Logit CTRR=ln {CTRR/(1-CTRR)}

Interaction†1: interaction between thin- and thick-side WJDs

Interaction†2: interaction between thin-side WJDs and RF and/or CCP

Interaction†3: interaction between thick-side WJDs and RF and/or CCP

Interaction†4: interaction between thin-, thick-side WJDs and RF and/or CCP

Analysis4: ANCOVA with the dependent variable and factors: age, RF and/or CCP, WJD thin-side, WJD thick-side, and interaction Interaction†1,2,3, and 4

Analysis5: ANCOVA with the dependent variable and factors: age, RF and/or CCP, WJD thin-side, WJD thick-side, and interaction Interaction†2

Analysis6: ANCOVA with the dependent variable and factors: age, RF and/or CCP, WJD thin-side, and WJD thick-side

F: F-value

df: degrees of freedom, expressed as F (df1, df2), where df1 is df for the factor between-groups and df2 is df for the error within-groups.

*: significant, P-value<0.05

**: significant, P-value<0.01
