# Supplementary material for: Asymmetrical Damage of the Wrist Joint Induces Lateralized Cortical Bone Loss in the Metacarpal Diaphysis in Patients with Rheumatoid Arthritis
Source: J Clin Med. 2024 Dec 16;13(24):7652. doi: 10.3390/jcm13247652 (PMC11676186; doi:10.3390/jcm13247652)
Supplement: Supplementary file 1 [file jcm-13-07652-s001.zip › Supplementary Data/Table S3.pdf]

Table S3. Analysis of covariance between Logit CTRR and associated parameters including prednisolone

		Analysis 7				Analysis 8		
		F	df	p-value		F	df	p-value
Logit CTRR	Age	0.091	1, 134	0.76		0.441	1, 137	0.51
	Prednisolone	0.001	1, 0.811	0.99		0.067	1, 137	0.80
	Thin-side WJD	1.774	1, 1.641	0.34		2.992	1, 1.014	0.68
	Thick-side WJD	0.222	1, 0.917	0.73		0.312	1. 1.004	0.68
	Interaction†1	17.420	1, 0.984	0.15		3.920	1, 137	0.05
	Interaction†2	7.828	1, 1.181	0.19		not included		
	Interaction†3	0.270	1, 0.977	0.70		not included		
	Interaction†4	0.250	1, 134	0.62		not included		

CTR: cortical thickness rate

In each patient, the side with the lower cortical thickness rate (CTR) of the metacarpals was designated the "thin-side" and that with the higher CTR the "thick-side."

WJD: wrist joint damage

CTRR: cortical thickness rate ratio= CTR (thin-side) / CTR (thick-side)

Logit CTRR=ln {CTRR/(1-CTRR)}

To approximate a normal distribution for analysis of covariance (ANCOVA), CTRR was transformed using logit transformation. This involved applying the logit function to CTRR values, defined as: Logit CTRR=ln {CTRR/(1-CTRR)}

Interaction†1: interaction between thin- and thick-side WJDs

Interaction†2: interaction between thin-side WJDs and Prednisolone

Interaction†3: interaction between thick-side WJDs and Prednisolone

Interaction†4: interaction between thin- and thick-side WJDs and Prednisolone

Analysis7: ANCOVA with the dependent variable and factors: age, Prednisolone, WJD thin-side, WJD thick-side, and interaction†1,2,3, and 4

Analysis8: ANCOVA with the dependent variable and factors: age, Prednisolone, WJD thin-side, WJD thick-side, and interaction†1

F: F-value

df: degrees of freedom, expressed as F (df1, df2), where df1 is df for the factor between-groups and df2 is df for the error within-groups.

*: significant, P-value<0.05

**: significant, P-value<0.01
