# Supplementary material for: Asymmetrical Damage of the Wrist Joint Induces Lateralized Cortical Bone Loss in the Metacarpal Diaphysis in Patients with Rheumatoid Arthritis
Source: J Clin Med. 2024 Dec 16;13(24):7652. doi: 10.3390/jcm13247652 (PMC11676186; doi:10.3390/jcm13247652)
Supplement: Supplementary file 1 [file jcm-13-07652-s001.zip › Supplementary Data/Table S4.pdf]

Table S4 Comparison of Laterality in Wrist Joint Damage and Prednisolone administration in RA Patients

	WJD Lateral (+)	WJD Lateral (-)	
Prednisolone (+)	14	59	73
Prednisolone (-)	10	60	70
	24	119	143

Prednisolone (+): Patients currently receiving prednisolone therapy.

Prednisolone (-): Patients not receiving prednisolone therapy.

WJD: wrist joint damage

WJD lateral (+) = WJD in one hand

WJD lateral (-) = WJD in both hands or no hand

WJD lateral (+) and Prednisolone (+) were not significantly associated according to the χ^2 test ($p = 0.43$)
