# Supplementary material for: Asymmetrical Damage of the Wrist Joint Induces Lateralized Cortical Bone Loss in the Metacarpal Diaphysis in Patients with Rheumatoid Arthritis
Source: J Clin Med. 2024 Dec 16;13(24):7652. doi: 10.3390/jcm13247652 (PMC11676186; doi:10.3390/jcm13247652)
Supplement: Supplementary file 1 [file jcm-13-07652-s001.zip › Supplementary Data/Table S5.pdf]

Table S5. Analysis of covariance between differences of thin-side CTR and thick-side CTR and duration of treatment with biologic agents

		Analysis 9		
		F	df	p-value
Differences of Thin-side CTR	Age	0.801	1, 24	0.38
	Duration#	0.697	1, 24	0.41
	Thin-side WJD	20.521	1, 22.816	<0.001
	Thick-side WJD	0.061	1, 24.687	0.81
	Interaction†[3]	0.01	1, 24	0.97
		Analysis 10		
		F	df	p-value
Differences of Thick-side CTR	Age	0.124	1, 24	0.73
	Duration#	1.126	1, 24	0.30
	Thin-side WJD	53.704	1, 1.966	0.019
	Thick-side WJD	24.050	1,2.795	0.019
	Interaction†[1]	0.023	1, 24	0.88

CTR: cortical thickness rate

In each patient, the side with the lower cortical thickness rate (CTR) of the metacarpals was designated the "thin-side" and that with the higher CTR the "thick-side."

WJD: wrist joint damage

Duration#: Duration of treatment with biologic agents

Interaction†: interaction between thin- and thick-side WJDs

Analysis9: ANCOVA with the dependent variable (Differences of Thin-side CTR) and factors: Duration#, WJD thin-side, WJD thick-side, and Interaction†

Analysis10: ANCOVA with the dependent variable (Differences of Thick-side CTR) and factors: Duration#, WJD thin-side, WJD thick-side, and Interaction†

F: F-value

df: degrees of freedom, expressed as F (df1, df2), where df1 is df for the factor between-groups and df2 is df for the error within-groups.

*: significant, P-value<0.05

**: significant, P-value<0.01
